# Dispersal Patterns, Active Behaviour, and Flow Environment during Early Life History of Coastal Cold Water Fishes

**DOI:** 10.1371/journal.pone.0046266

**Published:** 2012-09-28

**Authors:** Ryan Stanley, Paul V. R. Snelgrove, Brad deYoung, Robert S. Gregory

**Affiliations:** 1 Ocean Sciences Centre and Biology Department, Memorial University of Newfoundland, St. John's, Newfoundland, Canada; 2 Canada Research Chair in Boreal and Cold Ocean Systems, Memorial University of Newfoundland, St. John's, Newfoundland, Canada; 3 Department of Physics and Physical Oceanography, Memorial University of Newfoundland, St. John's, Newfoundland, Canada; 4 Ecological Sciences Section and Centre of Expertise for Aquatic Habitat Research, Fisheries and Oceans Canada, St. John's, Newfoundland, Canada; University of Plymouth, United Kingdom

## Abstract

During the pelagic larval phase, fish dispersal may be influenced passively by surface currents or actively determined by swimming behaviour. *In situ* observations of larval swimming are few given the constraints of field sampling. Active behaviour is therefore often inferred from spatial patterns in the field, laboratory studies, or hydrodynamic theory, but rarely are these approaches considered in concert. Ichthyoplankton survey data collected during 2004 and 2006 from coastal Newfoundland show that changes in spatial heterogeneity for multiple species do not conform to predictions based on passive transport. We evaluated the interaction of individual larvae with their environment by calculating Reynolds number as a function of ontogeny. Typically, larvae hatch into a viscous environment in which swimming is inefficient, and later grow into more efficient intermediate and inertial swimming environments. Swimming is therefore closely related to length, not only because of swimming capacity but also in how larvae experience viscosity. Six of eight species sampled demonstrated consistent changes in spatial patchiness and concomitant increases in spatial heterogeneity as they transitioned into more favourable hydrodynamic swimming environments, suggesting an active behavioural element to dispersal. We propose the tandem assessment of spatial heterogeneity and hydrodynamic environment as a potential approach to understand and predict the onset of ecologically significant swimming behaviour of larval fishes in the field.

## Introduction

The eggs and larvae of many marine organisms are transported during a pelagic dispersive stage through interaction between passive oceanographic processes [1], [2], and active behaviour [3]. Dispersal is essential to the maintenance of spatial structure [4] and stability of marine populations [5]. The success of the dispersal phase depends on avoiding predation, available food supply, and arrival at suitable nursery habitat. For the past century, ecologists and fisheries biologists have struggled to elucidate passive and active contributions to larval dispersal [6]–[9]. Though studies at relatively smaller scales of tens of kilometres, have resolved passive movement [10], empirical evidence of active behaviour in the field at broader scales remains elusive.

Multiple studies hypothesize that larval swimming ability is critical to success or failure in dispersal [11]. Laboratory studies illustrate potential contributions of swimming to spatial and temporal patterns in the field, and have shown that larval reef fish vary widely in their swimming and behavioural capabilities [9], [12], [13]. Recent laboratory swimming experiments demonstrate similar, though weaker, swimming capabilities in several cold ocean species, including Atlantic cod (*Gadus morhua*) [14]. Collectively, data from warm and cold ocean systems suggest that larval fish have the kinematic potential to influence their spatial distributions through active behaviour over a range of vertical and horizontal scales from metres to kilometres, potentially enhancing their capacity to select suitable habitat that can be vital to recruitment success [15].

Behavioural influences on spatial distributions of larvae are difficult to demonstrate without direct observations. Previous studies of coral reef systems [16], [17] include direct field observations of fish larvae that demonstrate swimming behaviour actively mediates dispersal trajectories. In cold ocean systems larval concentrations are often lower and larval durations more prolonged than those observed at lower latitudes, and therefore empirical field observations can be more difficult to obtain. Bradbury et al. [1] and Methven et al. [18] inferred aspects of swimming ability of larval and juvenile life history stages in cold ocean systems from ontogenetic changes in spatial distributions observed in the field.

Spatial distributions of marine organisms are rarely uniform and often patchy. Spatial patchiness of fish larvae is determined by passive oceanographic mechanisms [19], [20], survival [21], and active behaviour [22], [23]. Several studies on ichthyoplankton have noted analogous ontogenetic shifts in spatial distributions of larval fish [1], which in most cases is difficult to attribute to passive process alone. Because swimming capacity in larvae increases with size [9], [14], [24], and ontogeny parallels changes in spatial structure, past studies have linked heterogeneity in marine systems with active behaviour [20], [21], [23].

Previous patchiness studies do not specifically address the role of the hydrodynamic environment and how it might provide another framework in which to consider swimming as a quantifiable influence on spatial heterogeneity during the larval period. Researchers have used functional morphology to explain allometric growth trajectories in relation to hydrodynamic environments [25], and then often invoke the hydrodynamic environment to explain differences in the initial onset of substantive swimming behaviour in warm (at 5–8 mm, [13]) and cold water systems (at ∼10 mm, [1], [14]). Inefficient swimming characterizes small, slow-moving larvae, where viscous drag dominates, in contrast with the reduced viscous drag and increased swimming efficiency experienced by larger larvae in intermediate or inertial hydrodynamic environments [9], [26]–[28]. Because many pelagic larvae hatch at small sizes they are treated as passive organisms during the pelagic phase [19], [29]. Considering the close relationship between swimming efficiency, behaviour, and the hydrodynamic environment [13], [27], increased spatial heterogeneity towards the end of the larval period [1] likely coincides with the transition from an inefficient hydrodynamic environment to one more conducive to swimming. In larval fish, swimming behaviour can take many forms including vertical or diel movement [15], horizontal migrations [30], or a combination of both. All of these behaviours influence dispersal trajectories at some level [15] and all are subject to the constraints imparted on them by the physical hydrodynamic environment in which they operate.

Recent laboratory [14] and field [1] work on the larval stages of cold water species in Newfoundland suggests that swimming might play a significant role in dispersal and thus influence population structure through recruitment [1]. The productive bays in Newfoundland often provide key nursery areas for species such as Atlantic cod and capelin [31]–[33]. Advection from these regions may increase mortality of both larval and juvenile stages. The onset of active behaviour has been shown to help retain larvae in inshore habitats, and the onset of active behaviour thus defines a key life history influence on survival and recruitment [34], [35]. Our work builds on previous studies [29], [36] that argue for the importance of the timing of development of swimming behaviour at within-embayment scales of 10 s km, by uniting hydrodynamic theory and changes in spatial patchiness through larval ontogeny in the context of the larvae's physical and biological environment.

Smith Sound, Trinity Bay supports a large, persistent inshore spawning aggregation of Atlantic cod [37]. This aggregation provides a discrete, natal source of larvae to evaluate the potential contribution of larval swimming behaviour to cod dispersal. Trinity Bay is also characterized by high abundances of larvae of several other taxonomic orders with contrasting morphologies and ontogenies, including *Scorpaeniformes*, *Plueronectiformes. Perciformes* and *Osmeriformes*. This mix of taxa provides additional opportunities to test potential swimming contributions to spatial heterogeneity. We compare changes in spatial patterns of multiple species to passive flow conditions while simultaneously considering the hydrodynamic swimming environment and biological environment to which these larvae occupy. Specifically, we evaluate changes in spatial pattern related to swimming capability in order to test our hypothesis that increases in spatial patchiness coincide with critical changes in the hydrodynamic swimming environment of sampled larvae, thus helping to explain natural patterns in ichthyoplankton catch data.

## Materials and Methods

**Figure 1:**
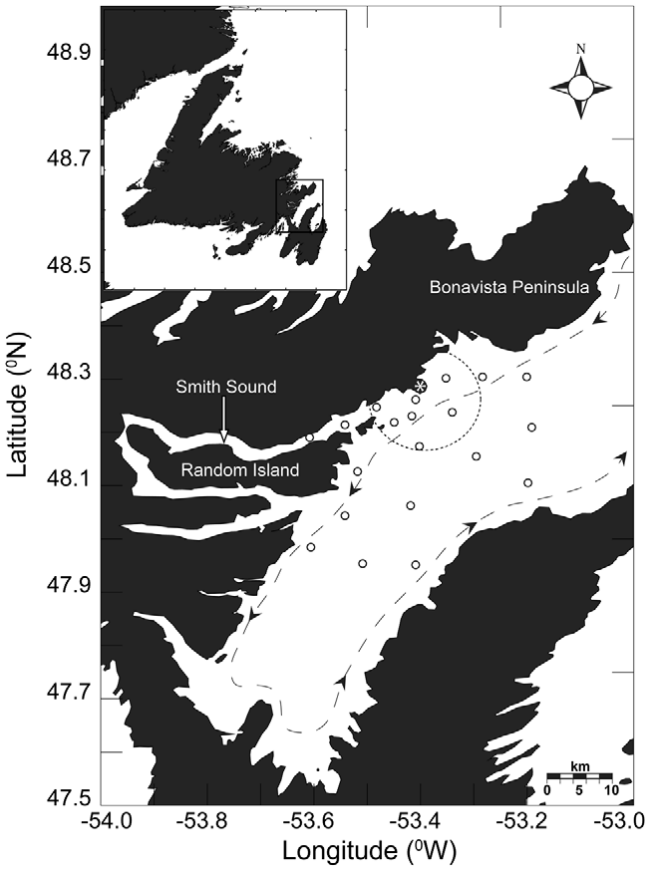
Ichthyoplankton sampling array. Trinity Bay ichthyoplankton survey array (O) sampled in May 2004/2006 and July 2004. Inset shows position of Trinity Bay relative to Newfoundland and Labrador. Dashed ellipse denotes boundary of a 7 km radius from Bonaventure Head (*). Dashed line with arrows represents mean flow conditions and circles represent sample stations.

We collected larval data from ichthyoplankton surveys of Trinity Bay, Newfoundland during the spring of 2004 and 2006, and the summer of 2004, on board the Canadian Coast Guard Ship (CCGS) Shamook. At each survey station tows were carried out using a 2.0 m by 2.0 m Tucker trawl fitted with decreasing mesh sizes of 1000, 570, and 333 m progressing from the front of the net to the cod end. We sampled twenty ichthyoplankton stations in a “bullseye” pattern radiating out from Smith Sound on the western side of the bay (Fig. 1) in oblique hauls to ∼40 m depth that encompassed the mixed layer [10], [38], which contains greater than 95% of the ichthyoplankton [39]. The Tucker trawl minimizes variability in catch estimates relative to other ichthyoplankton gear types [40]. Although size and species-specific net avoidance could not be measured directly, the large net size and sample volume (∼2000 m^3^) collected by the Tucker trawl is designed to minimize such biases. We preserved samples in a buffered seawater and 4% formalin solution, and later identified all fish larvae to species using published keys [41]. All necessary permits for collecting ichthoplankton were obtained prior to sampling in accordance with the Canadian Council of Animal Care guidelines. No specific locational permits were required for ichthyoplankton sampling in Trinity Bay, and no threatened or endangered species were at risk of incidental capture.

### Observed patchiness

Larvae were imaged at the Ocean Science Centre's Image Data Analysis Facility, where we measured lengths of a maximum of 100 individuals for each species for each station. Samples with more than 100 individuals of a single species were sub-sampled using a Motoda plankton splitter. We calculated length from the images to the nearest 0.1 mm with a pixel mm^−1^ calibration using ImageJ® image analysis software, and grouped larvae into 1 mm size bins. Standard length was defined as the measurement from the anterior tip of the body to the midlateral posterior edge of the hypural plate.

Linear kriging in Surfer® 8 was used to increase spatial resolution of surveys by using all stations sampled [42] to contour total abundances among sample stations. We interpolated and compared data on the two sides of the bay based on preliminary examination of kriging plots which suggested strong abundance differences between the eastern and western sides of Trinity Bay.

We used Lloyd's index of mean crowding [43] to determine spatial heterogeneity of larvae of different size classes in the sample grid (Fig. 1). Lloyd's index quantifies the occurrence of an individual in a given sample relative to an individual from a randomly distributed population with the same mean density [43]. Because the calculation is independent of larval concentration, it is particularly useful for comparing among years, areas, or developmental stages and has therefore been widely used in marine patchiness studies [1], [18], [20], [23].

The calculation of Lloyd's index requires an estimate of population variance where the use of sample variance may be inappropriate. Several authors estimated population variance by applying a negative binomial distribution to ichthyoplankton data [20], [23], [43] according to methods outlined by Bliss and Fisher [44]. We utilized a negative binomial distribution to apply a maximum likelihood expression [44] to the station count data, as used in similar studies [20], [23], [43], to estimate population variance. We verified the effectiveness of this estimation method using data from Matsurra and Hewitt [23], and found it consistent with their estimates of population variance in all comparisons. We then incorporated the estimate of population variance (*k*) into Lloyd's index of patchiness [1], [18], [45], simplifying Lloyd's index equation to:
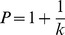
(1)where *P* is an estimate of patchiness and *k* is the dispersion parameter estimated with the maximum likelihood approach [44]. Estimates of *P* greater than 1 denote how many more times crowded an individual is than a random distribution [43]. Standard error was estimated following methods outlined in Lloyd [43] and later studies [1], [23].

### Hydrodynamic environment

For each larval fish measured we calculated the Reynolds number (*Re*) following Brett [46]

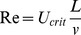
(2)where *U_crit_* = critical swim speed (m s^−1^), *L* is the length scale and ν is the kinematic viscosity of seawater (m^2^ s^−1^). We used total length (*L*), defined as the mean size within a 1 mm size bin and applied this mean length to the swimming parameter (U_crit_) of the Reynolds number equation. Critical swim speeds for Atlantic cod were estimated according to length swimming relationships derived from an image database and corresponding swim data reported in Guan et al. [14]. Critical swim speeds for the remaining larvae were estimated using a statistically significant multispecies length-swimming model (R^2^ = 0.90) presented by Guan et al. 2008. The consistent functional relationship between swimming capacity, beat frequency, and length in larval fish suggests some generality among temperate species at comparable ontogenetic stages [1], [24]. Although Guan et al. [14] based their equation on species that only partly overlap our focal species, we believe that the strong fit of their multispecies regression suggests a consistent pattern across species that offers the best available approximation for critical swimming capacity in these species.

We obtained measurements of average mixed layer (upper 40 m) temperature, salinity, and fluorometry from vertical conductivity-temperature-depth (CTD) profiles taken concurrently with each ichthyoplankton sample. Kriging was used to analyze spatial patterns of environmental variables which we then compared with spatial patterns of larvae produced the same way. Kinematic viscosity (ν) was calculated from mean survey temperature data collected from CTD casts. Kinematic viscosity also changes as a function of salinity, however, average salinity differences among survey seasons were negligible (maximum difference of 0.7), and we therefore did not consider them.

We estimated Reynolds number for a specific size range of larvae based on swimming velocities from the morphometric kinematic relationships and average kinematic viscosities. Estimates of the transitional point between swimming environments, defined by *Re*, varies among studies. We used a Reynolds number of 300 as a critical point marking the shift between a clearly viscous swimming environment to one which is intermediate [13], [26] or even inertial [25].

We estimated both Lloyd's index and Reynolds numbers for larvae collected in the field using length as the linking factor in all calculations. Information on the hydrodynamic environment, defined by Reynolds number boundaries, was then considered with patchiness data to illustrate linkages between hydrodynamics and spatial heterogeneity in the field.

### Biological environment

**Figure 2:**
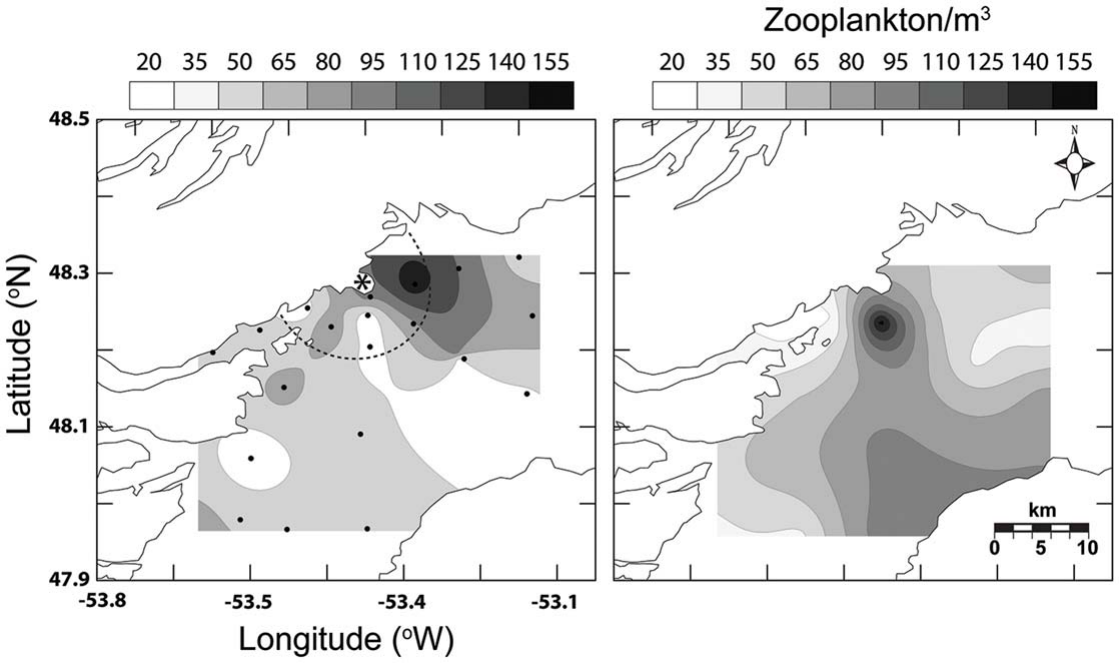
Spatial pattern of zooplankton abundance. Spatially contoured zooplankton abundance (individuals per m^3^) collected during May 24–26 (*A*) and May 29–31 (*B*) 2006 plankton surveys [47]. Dots denote actual sampling locations. Dashed ellipse denotes boundary of a 7 km radius of Bonaventure Head (*).

Finally we used information collected during the ichthyoplankton surveys to characterize the environment that larvae occupy. Specifically samples from May 2006 were processed for zooplankton abundance [47], which identified specimens to species where possible and sub-sampled to a minimum count of 300 individuals•sample^−1^ using a Motoda plankton splitter. We then mapped and compared zooplankton abundance following the same methodology outlined for larval fish lengths (Fig. 2). Temperature and fluorescence data from CTD deployments provided spatial information on the physical environment and primary production of the bay. Combined these metrics provide a parallel assessment of the environment experienced by the larvae throughout ontogeny, providing a possible framework to describe larval spatial patterns.

## Results

### Observed patchiness

**Figure 3:**
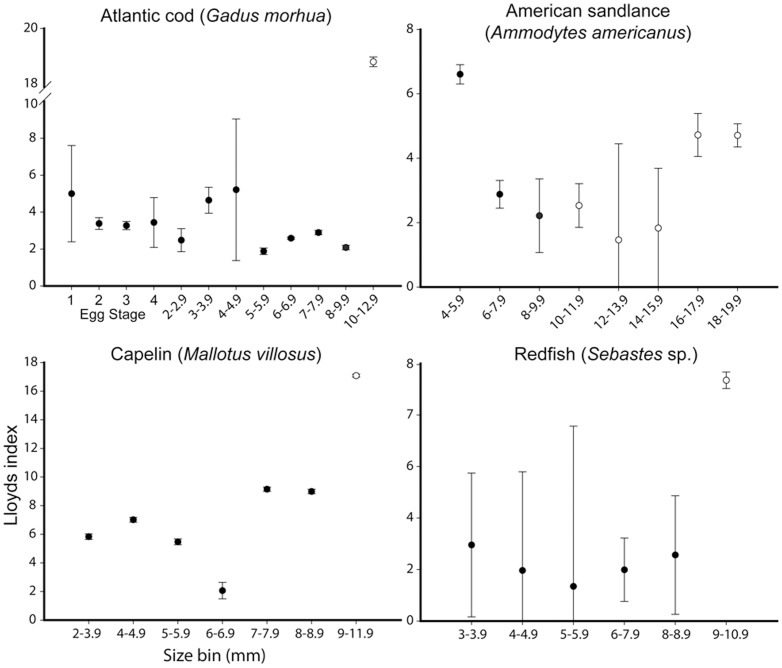
Patchiness through ontogeny: cod, sandlance, capelin and redfish. Spatial patchiness of larvae presented as Lloyd's index of patchiness ±1 standard error. Viscous and intermediate-inertial larvae are differentiated by filled and open circles. Larval data is pooled from all ichthyoplankton surveys (May 2004, 2006 and July 2004) as a result of constraints on catch numbers and Lloyd's index calculation.

**Figure 4:**
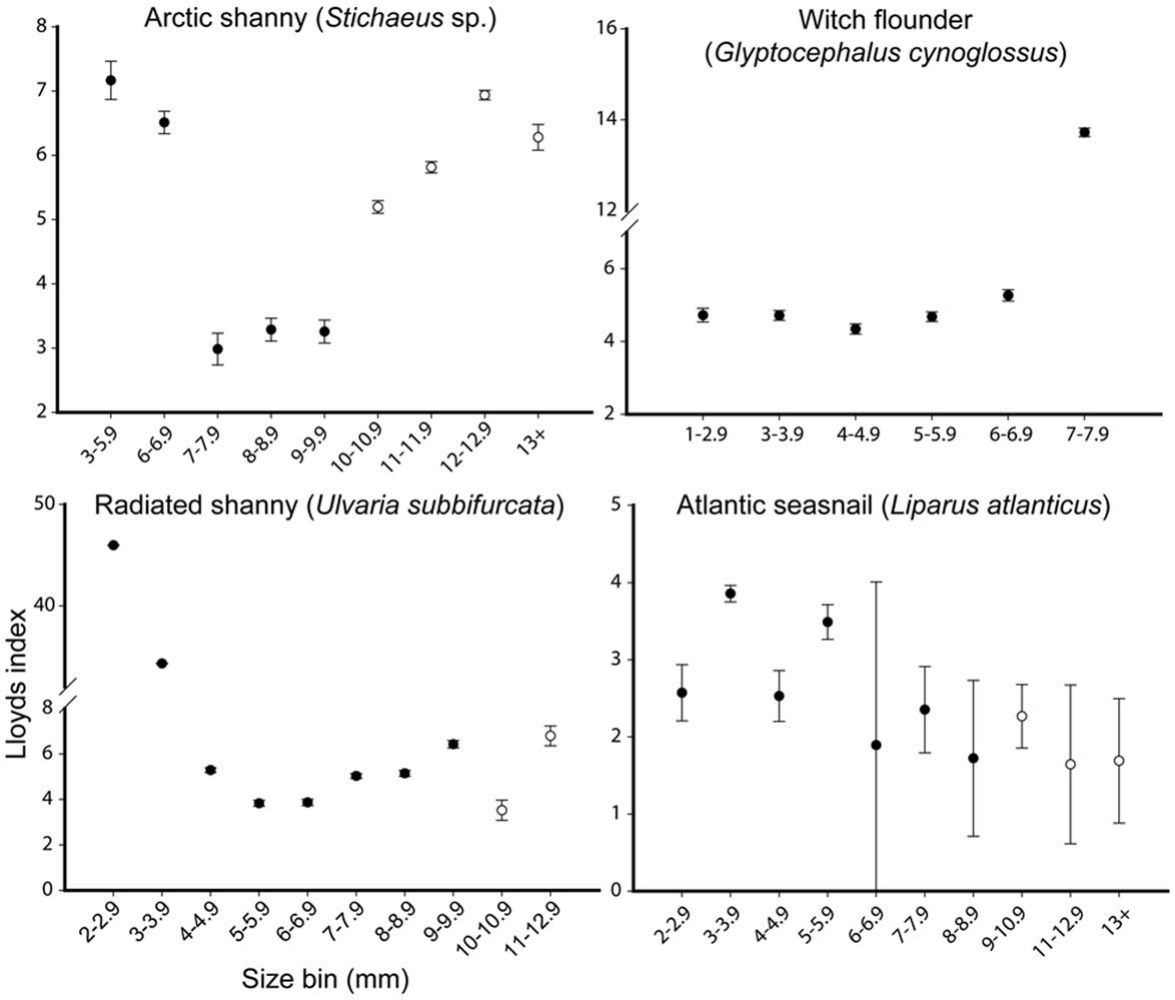
Patchiness through ontogeny: arctic shanny, witch flounder, radiated shanny and seasnail. Spatial patchiness of larvae presented as Lloyd's index of patchiness ±1 standard error. Viscous and intermediate-inertial larvae are differentiated by filled and open circles. Larval data is pooled from all ichthyoplankton surveys (May 2004, 2006 and July 2004) as a result of constraints on catch numbers and Lloyd's index calculation.

We limited our spatial analysis of Lloyd's index as a function of the full range of ontogenetic egg development to Atlantic cod, which was the only species in our samples with sufficiently abundant developmental stages to include in patchiness estimates. Nonetheless, comparisons of larval patterns for multiple species indicated consistent patterns of patchiness through larval ontogeny (Fig. 3,4). For Atlantic cod, patchiness in eggs and early larvae were similar. We observed increased patchiness in the largest larval size grouping of all 8 species examined except for radiated shanny (*Ulvaria subbifurcata*) and Atlantic seasnail (*Liparus atlanticus*) (Fig. 4). The standard error estimated about each mean varied from 0.05 in witch flounder (*Glyptocephalus cynoglossus*) to 3.2 in Atlantic seasnail. The effect of zero counts (stations where no larvae of a given species at any size were caught) differed among species, exaggerating patchiness estimates for species with abundant larvae (e.g. capelin) or reducing estimates for species with few larvae (e.g. redfish). Despite this variability, the overall pattern with size was similar irrespective of whether we included zero values. To be consistent with other authors who used this method [1], [20], [45], we removed all zero stations from our analyses.

**Figure 5:**
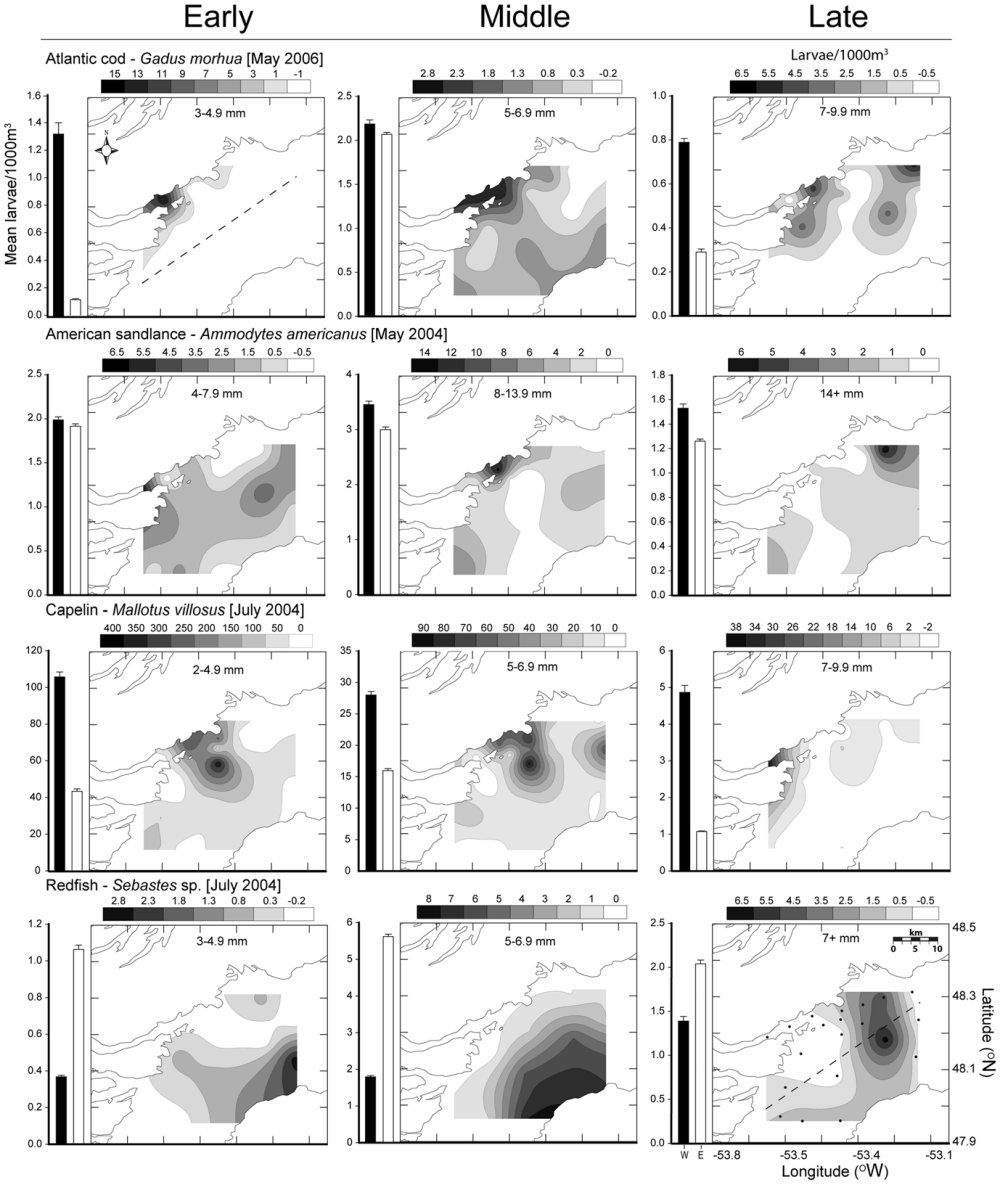
Spatial pattern of larval abundance: cod, sandlance, capelin and redfish. Spatially contoured species size abundance binned into early, middle, and late larval developmental sizes. Side bars represent mean larval abundance for western (W, black bar) and eastern (E, white bar) regions of Trinity Bay. Dashed line represents the boundary between the sample regions and dots represent sample stations.

**Figure 6:**
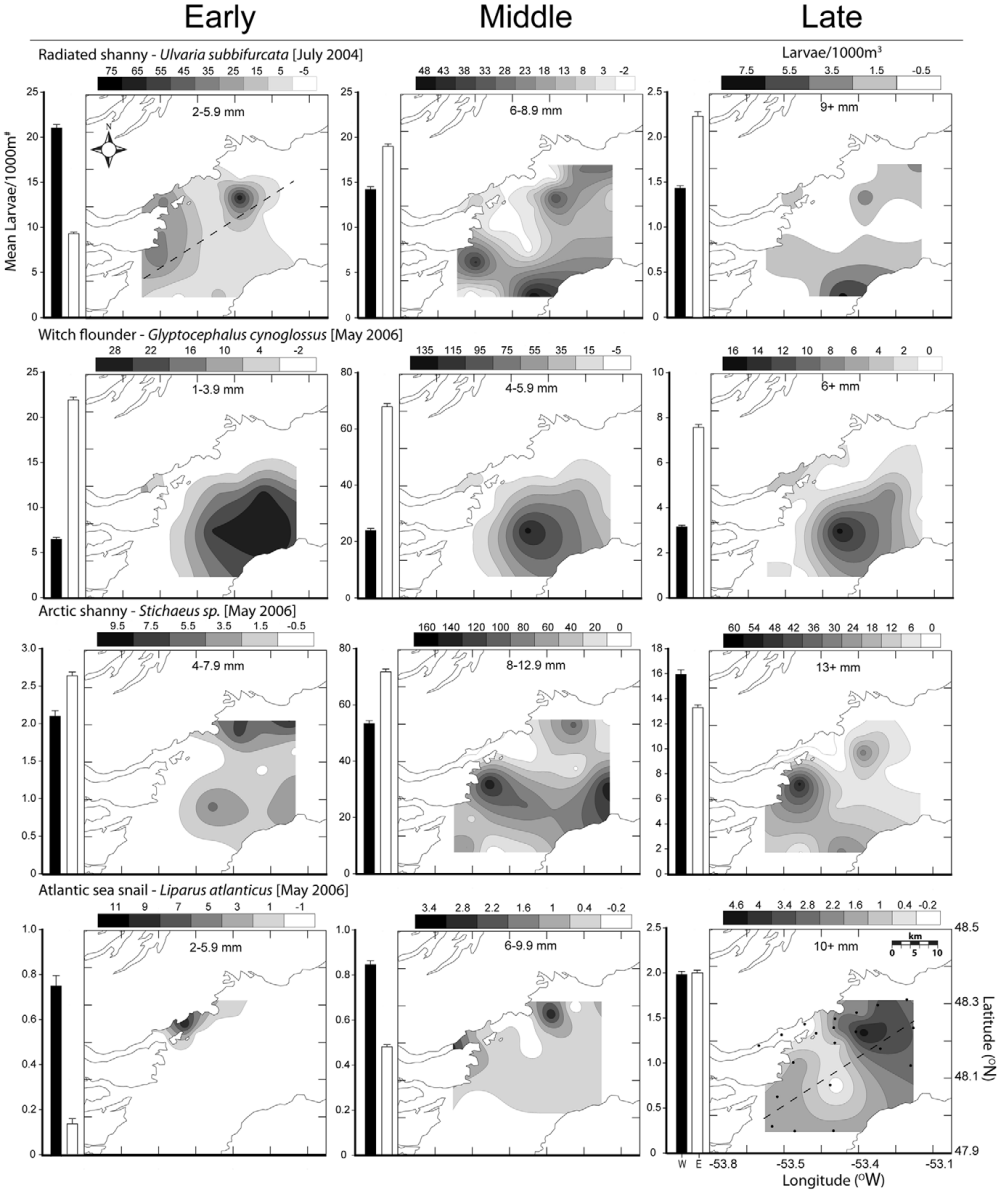
Spatial pattern of larval abundance: arctic shanny, witch flounder, radiated shanny, and seasnail. Spatially contoured species size abundance binned into early, middle and late larval developmental sizes. Side bars represent mean larval abundance for western (W, black bar) and eastern (E, white bar) regions of Trinity Bay. Dashed line represents the boundary between the sample regions and dots represent sample stations.

Spatial heterogeneity as a function of larval ontogeny for larvae of 8 species suggested that patterns in early and late larval stages are more defined than in intermediate stages (Fig. 5,6), as indicated by the patchiness analysis. Redfish (*Sebastes* spp.), radiated shanny, and witch flounder were all significantly more abundant on the eastern side of Trinity Bay throughout larval ontogeny. Arctic shanny (*Stichaeus puntatus*.) and Atlantic seasnail shifted between the east and west divide during the latest larval stage. The remaining species were significantly more abundant on the western side of Trinity Bay throughout ontogeny. Larger individuals of Atlantic cod, American sandlance (*Ammodytes americanus*), capelin (*Mallotus villosus*), redfish, and Atlantic seasnail were more common on the western side of Trinity Bay near Bonaventure Head.

Kriging smoothed out bias in the sampling grid, such as our highly clustered stations near the presumed natal source of cod larvae in Smith Sound. This procedure provides a reasonable estimate of mean concentration but an unreliable estimate of variance for each side of the bay because variance estimates for the east and west side of the bay are not independent. To determine if this statistical violation impacted spatial interpretation, we divided the bay into western and eastern regions, and individually kriged data on Atlantic cod and American sandlance (Fig. 5). With this approach we could compare means without the problem of non-independent variance. In all tests, the means and variances changed by less than 5% and in no case did the interpretation of results change. Although the analyses we present here defy the assumption of independent variance, we infer from this secondary comparison that the results are meaningful and provide a reliable analysis of spatial patterns, despite spatial bias in sampling stations.

### Hydrodynamic environment

For Atlantic cod, of all morphometric variables analysed, total length was the best predictor of kinematic potential [35], as shown in previous studies [14], [24], [48]. This finding supports the use of larval length in Reynolds number calculations. Reynolds number was calculated using critical swim speeds (U_crit_) for Atlantic cod larvae of a given total length (TL) derived from:
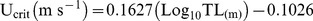
(3)Because we lacked the data to develop similar critical swim speed equations for other species we used the best available multi-species swimming model presented by Guan et al. [14] derived from local species and environmental conditions.

We calculated kinematic viscosity from the mean temperature for the mixed layer (top 40 m) at all stations during all surveys. Although this method ignores spatial variability in viscosity, extreme high and low field temperatures change the estimate of Re by less than 3.5%, and therefore have little effect on data interpretation. Mean temperature among surveys yielded an average kinematic viscosity of 1.714×10^−6^ m^2^ s^−1^ (SD = 0.138, N = 6).

We found similar patterns of patchiness in the context of hydrodynamic transitions among species by calculating the mean change between the size classes for each species immediately before and after the proposed viscous transition (Re = 300). For all species except Atlantic seasnail (decrease 10%), an increase in patchiness coincided with the lead up to and transition out of the viscous environment (range of ∼10–300% increase with a mean of 255% (Fig. 3,4). Size classes sampled for witch flounder did not attain Reynolds numbers >300, though spatial heterogeneity increased at the largest size classes sampled which approached the proposed transitional size. Variability in patchiness estimates also typically decreased after this critical transition (Fig. 3,4). Atlantic seasnail patchiness decreased throughout ontogeny with later sizes approaching random or uniform distribution (Lloyds index = 1).

### Physical and biological environment

All data collected from CTD survey profiles showed colder water on the western side of Trinity Bay near Bonaventure Head than the bay mean (Table 1). On average, the area immediately surrounding Bonaventure Head was 0.5°C cooler than the survey average for both surface- and mixed-layer measurements. Inter-annual or seasonal differences in mixed layer temperature were minimal. Measures of primary production represented by chlorophyll “*a*” concentrations were significantly higher on the western side of Trinity Bay compared to the eastern side in all surveys, in particular within the area immediately surrounding Bonaventure Head (Table 1). Similarly, May 2006 survey data indicated a strong spatial association of zooplankton abundance (individuals m^−3^) with the western side of the bay near the cold upwelling (Fig. 2.). Larger individuals of Atlantic cod, American sandlance, capelin, redfish, and Atlantic seasnail also demonstrated a spatial association with this area of heightened productivity (Fig. 5,6).

**Table 1:** East-west comparison of biological and physical characteristics of Trinity Bay.

Variable	Factor	df	*F*-value	p-value
Fluorescence	Side of bay	1	10.420	0.002
	Survey	6	3.957	0.001
	Interaction	6	3.699	0.002
Mixed Layer Temperature	Bonaventure Head	1	12.166	0.001
	Survey	6	32.927	<0.0001
	Interaction	6	0.322	0.925
Zooplankton*	Bonaventure Head	1	6.901	0.013
	Survey	6	8.866	0.005
	Interaction	6	0.694	0.410

Results for General Linear Model Analysis of Variance comparing physical and biological parameters between sides of Trinity Bay (fluorescence) and between survey average and upwelling (stations within 7 km of Bonaventure Head).

* Zooplankton data only available for 2006 surveys.

## Discussion

Understanding the processes underlying the spatial dynamics of a population is invaluable for fisheries management [49]–[51] and marine conservation design [2], [52]. For many fish species significant movement and connectivity is achieved during the larval phase through dispersal, which represents a combination of both passive and active processes [9]. Our study provides several lines of evidence that larval behaviour influences dispersal trajectories, and that the onset of this behaviour fits predictions based on hydrodynamic transitions to more favourable swimming environments.

Analysis of spatial heterogeneity as a function of larval length suggests that swimming ability plays an increasingly important role through larval ontogeny. Ontogenetic shifts in spatial pattern, and especially the increased patchiness reported at larger larval size classes by several authors [20], describe the majority of species we collected in Trinity Bay (Fig. 3,4). Most importantly, patchiness increased at the largest sizes of all taxa of larvae we sampled, with the lone exception of Atlantic seasnail. Passive advection of eggs and small larvae living in a viscous environment [11] coupled with the discrete nature of adult spawning [22] explain high spatial patchiness for small larval sizes. This trend was evident for Atlantic cod in our study and others [1], where patchiness was similar in early larval stages and eggs. If passive processes alone dictate spatial pattern, there would be no reason to expect the consistently high patchiness we observed in early and late stage larvae and low patchiness in intermediate-sized larvae of multiple species.

Larval spatial distributions in Trinity Bay changed with larval size and thus development. Snelgrove et al. [29] noted that the late larval stages of several species in Placentia Bay, Newfoundland occurred in areas of elevated productivity that were inconsistent with a passive model of dispersal given the ambient flow conditions in that bay. They argued that larval peak abundances were ‘upstream’ of predicted location based on advection, and active behaviour was therefore necessary to explain spatial pattern. In our study, Atlantic cod, American sandlance, capelin, and Atlantic seasnail were all associated with the western side of Trinity Bay throughout larval ontogeny, as were highest abundances of late stage redfish and Arctic shanny. Only witch flounder and radiated shanny larvae were not strongly associated with western Trinity Bay; witch flounder were actually associated with the east coast of Trinity Bay, and showed no evidence of displacement with ontogeny. Although Tucker trawls integrate over the upper 40 m mixed layer and therefore do not allow us to address the role of vertical shear in creating these patterns [15], the very nature of a mixed layer suggests that this strategy may add noise but not bias.

Spatial patterns in larval fishes were inconsistent with a passive dispersal model, given mean bay-scale currents. Circulation modelling by Yao [53] and Tittensor et al. [54], [55] illustrate that an inshore branch of the Labrador Current strongly influences circulation patterns in Trinity Bay. Current meter observations indicate mean flow direction typically equals the variance [54], [55], although there is a clear mean circulation pattern of water entering on the west and exiting on the eastern coast. Flow variability, primarily driven by wind stress, produces patterns of current response dominated by a complex upwelling-downwelling cycle and a Kelvin wave response [56]. A counter-clockwise gyre near the mouth of Smith Sound produces some of the strongest currents in Trinity Bay [55]. Passive residency times in Trinity Bay, based upon particle tracking calculations made using the CANDIE circulation model [56], are on the scale of days to weeks [54], [55]. Larvae of all species, with the exception of radiated shanny and witch flounder, were “upstream” of locations predicted by passive processes alone, suggesting a non-passive dispersal component. The static spatial distribution of witch flounder in our study was also inconsistent with a passive model. Swimming ability has been shown to scale positively with length for numerous fishes [14], [24]. Therefore, the ‘upstream’ distribution of larger larvae we observed supports the suggestion that active larval behaviour could influence spatial pattern.

The spacing of our sampling grid varied from 2 km to 5 km. Although Lloyd's index of mean crowding does not incorporate a spatial component [43] we must consider the spatial representation of our sampling grid. Previous authors utilized the steady state approximation of the advection-diffusion equation in order to estimate behavioural capacities needed to maintain patch structure:
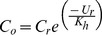
(4)where C_o_ is concentration at patch origin, *C_r_* is the concentration at patch perimeter, *U_r_* is the swim speed of the larvae and *Kh* is the horizontal diffusivity of the system [20]. Using this equation we solved for the swim speed needed to maintain 99% of patch structure. Given known horizontal diffusivity estimates calculated in coastal Newfoundland waters range from 70–100 m^2^ s^−1^ [57] we estimate that swimming speeds of at least 1–1.2 cms^−1^ are needed to maintain patches in our system. Given the relationships demonstrated with the multispecies and cod length swim speed models and a more basic body length argument (see Bradbury and Snelgrove [8]), the upper size ranges of larvae we sampled have the swimming capacity necessary to maintain the observed patch structure based on the steady state approximation.

The hydrodynamic environment imparts physical limitations on an organism's behavioural capacity by restricting kinematic potential, especially for small individuals [26], [58]. In general, behavioural capabilities of larval fishes are expected to differ among species and orders [59], [60], as reflected in our data on the magnitude, direction and among-species differences in fit of ontogenetic-patchiness patterns. Though variable among species, a consistent trend of higher patchiness estimates at early and later larval sizes nonetheless emerges. Integration of morphometric, kinematic, and spatial analyses allows interpretation of observed distributions of larvae in the field, in light of their hydro-mechanical constraints. Increased spatial heterogeneity at or near the transition from the viscous to more favourable hydrodynamic environments suggests active swimming behaviour plays a role in larval distribution patterns. The transition to intermediate-inertial regimes corresponded to increased spatial patchiness for all species except Atlantic seasnail, where low numbers of individuals limited our confidence in patchiness estimates. For Atlantic cod, capelin, redfish, and arctic shanny the transition from a viscous environment (∼Re>300) coincided with a 3–8 fold increase in patchiness. These species were strongly associated with western Trinity Bay. Although we observed no marked increase in patchiness in American sandlance and radiated shanny during the transition to the inertial environment, their patchiness nonetheless increased after the transition as predicted with active swimming.

Results from our hydrodynamic spatial pattern analysis were strikingly similar to conclusions drawn by other studies on spatial patchiness and critical points in larval development. Several studies identify 10 mm as a critical ontogenetic benchmark in terms of behaviour and spatial pattern observed in the field for multiple species, including larval capelin [39], walleye pollock [20], Atlantic cod [1], [14] and shorthorn sculpin [14]. Our analyses suggest a transition length of 9.3 mm for Atlantic cod and 9.5 mm for the other species we examined. The 10-mm threshold [1], [39] might be partially explained by the critical hydrodynamic shift suggested by our study. Studies of fish in warmer, and therefore less viscous, waters indicate an earlier transition (5–8 mm) [13] to an inertial flow environment. For example, the ∼15°C difference between coral reef waters and those in coastal Newfoundland correspond to a ∼45% decrease in viscosity. The swimming comparison between different size at hatch among species reported by Guan et al. [14] and our field study on spatial distributions provide complementary lines of evidence that larval behaviour could contribute to local dispersal trajectories and possibly to larger-scale population structure in cold ocean environments.

Adult spawning location clearly plays a role in setting initial patterns for early egg and larval stages, and our data set is inadequate to fully evaluate how these behaviours contribute to such patterns. Nonetheless, although abundances of early stage eggs of pelagic spawners and small larvae of demersal spawners offer insights into spawning locations [29], our study focuses primarily on contrasting how distribution of ontogenetic stages change as ontogeny progresses. Knowledge of spawning locations is therefore unnecessary.

The results presented above indicate that passive circulation alone cannot explain spatial distribution pattern, leaving two possible explanations for our results other than active swimming - predation or food availability. Size-dependent predation in particular could influence spatial patterns in ichthyoplankton. Several studies of larval distribution in Newfoundland embayments identified juvenile or adult capelin (*Mallotus villosus*) as the dominant predators of larval fish [1], [49], [61], [62]. Previous acoustic work found juvenile and adult capelin consistently associated with the more productive western side of Trinity Bay [49]. If predation drives larval patchiness, we would expect size-dependent mortality and lower larval abundances on the western side of the bay. The observed species distributions and abundances indicate that mortality and predation alone cannot explain patterns in patchiness. Although predation cannot be fully dismissed in our study, patterns in patchiness are more easily explained by active swimming behaviour.

Food availability can also concentrate organisms into patches [43] or lead to mortality patterns, either through direct starvation or increased vulnerability to predators. In Conception Bay, adjacent to Trinity Bay, previous work showed that larval fish consume less than 0.1% of available microzooplankton, suggesting that food limitation is unlikely in this ecosystem [63]. Although predator-prey encounter rates can influence larval growth and survival [64], heightened chlorophyll and zooplankton concentrations associated with persistent upwelling on the western side of Trinity Bay near Bonaventure Head [49], [53]–[55], [65] suggest abundant microzooplankton and copepod eggs, which are preferred food by larval fish [63].

Using behaviour to explain spatial pattern necessitates the resolution of bearing or frame of reference [66]. Our analysis suggests a north-western association of larvae from multiple species and origins. For behaviour to influence distributions at the spatial scales measured (10 s of km), larval fish would likely use a variety of sensory cues, including auditory, olfactory or light cues [67]. Work with reef fish has shown that fish have the ability to differentiate and orient towards scent cues [68]. In Trinity Bay the spatially discrete nature of fluorescence and zooplankton abundance could provide a signal for directed movement to the western portion of Trinity Bay. Similarly, the north-western portion of Trinity Bay is at the mouth of Smith Sound which might produce a unique olfactory or possibly auditory signature, possibly explaining the association of multiple species with western Trinity Bay. The ability of larvae to follow gradients, and food gradients in particular, has been suggested as a possible dispersal cue [69]. The heightened primary productivity and zooplankton abundance on the western portion of Trinity Bay could similarly provide an orientation signal to multiple species.

Data from our study do not permit distinction of whether this behaviour is a response to single or multiple cues. However, our study does suggest that multiple species conform to a pattern fitting predictions of behaviour and not one predicted by a purely passive model. Increased swimming abilities both horizontally and vertically will enable larvae to utilize and orient towards a greater diversity of cues [67].

### Summary

Spatial analysis using total length as a proxy for ontological development revealed higher patchiness estimates for late stage and in some cases early stage larvae than at intermediate stages, confirming patterns found in other temperate species [1], [18], [20]. Spatial patterns of larval abundance revealed an association between, and in some cases progression towards, the western coast of Trinity Bay throughout ontogeny. Both of these patterns were inconsistent with predictions based on passive transport, indicating that patterns were not driven primarily by passive physical processes. The presence of late-stage larvae in productive upwelling at times when larvae transition from a viscous to inertial hydrodynamic environment suggests that the kinematic potential of larval fish as small as 9 mm can influence dispersal patterns and thus possibly population connectivity over larger scales. Determining whether this influence is achieved through actual horizontal movement or vertical movement (e.g. in response to tides) that modifies horizontal transport, and to what sensory cue this behaviour might respond to, would require in situ observations beyond the scope of our study. Nonetheless, the net result is swimming may produce substantial changes in distribution through ontogeny that may contribute to recruitment variation. This analysis is particularly relevant for the comparatively viscous environment experienced by cold ocean species sampled at larger spatial scales, which differs from the coral reef environments where most larval swimming studies are conducted over finer spatial scales.
